# Diffuse glioneuronal tumor with oligodendroglioma-like features and nuclear clusters (DGONC), new name and new problems: an illustration of one case with atypical morphology and biology

**DOI:** 10.1186/s40478-024-01822-y

**Published:** 2024-06-26

**Authors:** Arnault Tauziède-Espariat, Lelio Guida, Volodia Dangouloff-Ros, Nathalie Boddaert, Gaëlle Pierron, Delphine Guillemot, Julien Masliah-Planchon, Lauren Hasty, Alice Métais, Fabrice Chrétien, Pascale Varlet

**Affiliations:** 1 Department of Neuropathology, GHU Paris - Psychiatry and Neuroscience, Sainte-Anne Hospital, 1, rue Cabanis, Paris, 75014 France; 2 Institut de Psychiatrie et Neurosciences de Paris (IPNP), UMR S1266, INSERM, IMA-BRAIN, Paris, France; 3 Laboratory of Somatic Genetics, Université de Paris, France3 Curie Institute Hospital, Paris, Paris, France; 4 Department of pediatric Neurosurgery, APHP Hopital Universitaire Necker Enfants Malades, Université Paris-Cité, Paris, Paris, France; 5 Department of pediatric Radiology, APHP, Hôpital Universitaire Necker Enfants Malades, Paris, France; 6 Institut Imagine, INSERM U1299 and UMR 1163, Université Paris-Cité, Paris, France; 7 Paris-Sciences-Lettres, Curie Institute Research Center, INSERMU830, Paris, France; 8 Curie Institute Hospital, Laboratory of Somatic Genetics, Paris, France

**Keywords:** DGONC, Chromosome 14, PIK3R1, Embryonal

## Abstract

A novel histomolecular tumor of the central nervous system (CNS), the “diffuse glioneuronal tumor with oligodendroglioma-like features and nuclear clusters (DGONC),” has recently been identified, based on a distinct DNA methylation profile and has been added to the 2021 World Health Organization Classification of CNS Tumors. This glioneuronal tumor mainly affects the supratentorial area in children and recurrently presents with a monosomy of chromosome 14. Herein, we report the case of a DNA-methylation based diagnosis of DGONC having atypical features, such as pseudo-rosettes and the absence of a chromosome 14 monosomy, thus rendering its diagnosis very challenging. Because of the wide variety of morphologies harbored by DGONC, a large range of differential diagnoses may be hypothesized from benign to malignant. Interestingly, the current case, like one previously reported, exhibited a co-expression of OLIG2, synaptophysin and SOX10, without GFAP immunopositivity. This particular immunophenotype seems to be a good indicator for a DGONC diagnosis. The classification of DGONC amongst glioneuronal or embryonal tumors is still debated. The clinical (a pediatric supratentorial tumor), morphological (from a benign oligodendroglioma-like tumor with microcalcifications and possible neuropil-like islands to a malignant embryonal tumor with a possible spongioblastic pattern), and immunohistochemical (co-expression of OLIG2 and synaptophsyin) profiles resemble CNS, neuroblastoma, *FOXR2-*activated and may potentially bring them together in a future classification. Further comprehensive studies are needed to conclude the cellular origin of DGONC and its prognosis.

## Introduction

The “diffuse glioneuronal tumor with oligodendroglioma-like features and nuclear clusters” (DGONC) is a new tumor type recently isolated by a distinct DNA-methylation profile and introduced in the latest World Health Organization (WHO) Classification of Brain Tumors [1, 2]. These tumors frequently harbor a chromosome 14 monosomy [1, 2]. To date, 44 cases have been reported in the literature and very few data concerning their histopathological, radiological and clinical features are available [1, 3–5]. The question concerning its classification within the chapter of glioneuronal or embryonal tumors is still debated [2]. Herein, we report an atypical case of DNA-methylation proven DGONC without a chromosome 14 monosomy and histopathological features resembling a Central Nervous System (CNS) neuroblastoma, *FOXR2*-activated (CNS, NB-FOXR2).

## Case presentation

**Fig. 1:**
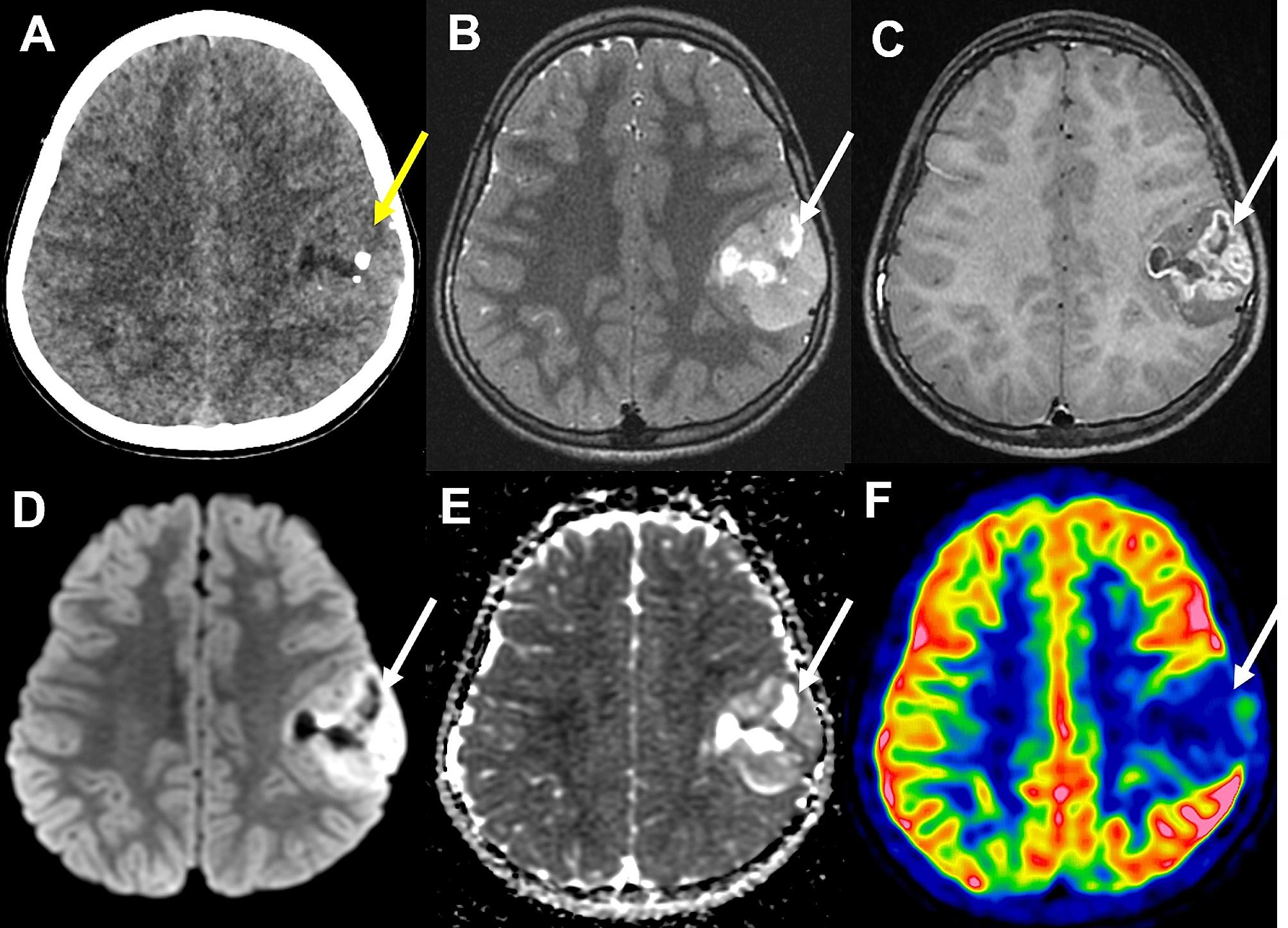
Radiological features of the tumor. (**A**) A computerized tomodensitometry scan showed a slightly hyperdense heterogeneous cortical mass with calcifications in the left central sulcus. Bone scalloping was observed next to the tumor. Magnetic resonance imaging showed the mass to have high signal intensity on T2-weighted images (**B**), heterogeneous contrast enhancement (**C**), high signal intensity on diffusion weighted images (**D**) with intermediate apparent diffusion coefficient (**E**) and low cerebral blood flow by arterial spin labeling perfusion (**F**)

**Fig. 2:**
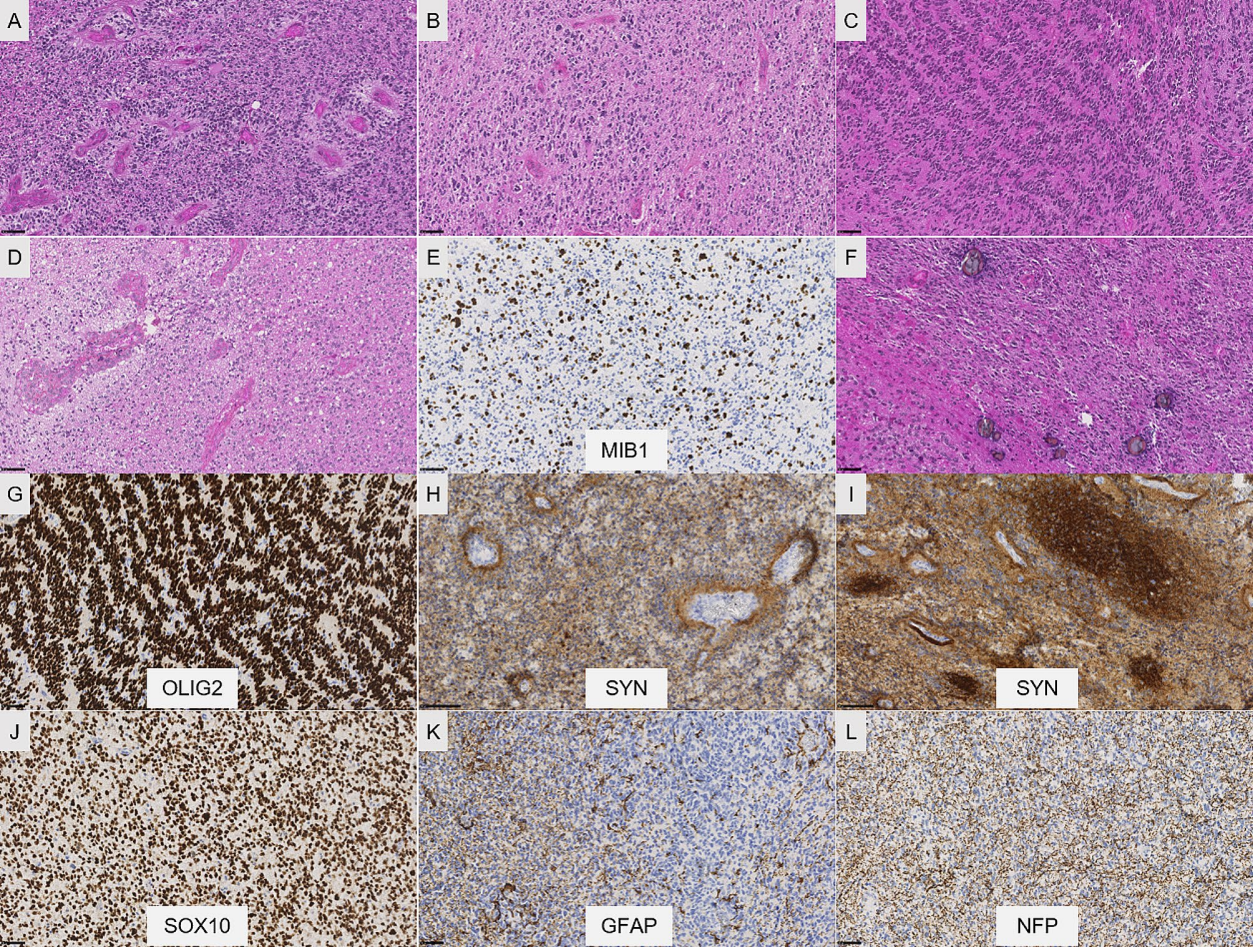
Histopathological features. (**A**) A highly cellular tumor showing varied histopathological patterns composed of pseudorosettes (HPS, magnification 400x), oligodendroglioma-like features and nuclear clusters (HPS, magnification 400x) (**B**) and spongioblastic arrangements (HPS, magnification 400x) (**C**). Microvascular proliferation (HPS, magnification 400x) (**D**) and microcalcifications (**E**) were present (HPS, magnification 400x). MIB1-labeling index was high (magnification 400x) (**F**). Diffuse expression of OLIG2 by tumor cells (magnification 400x) (**G**) and synaptophysin with perivascular strong staining (magnification 400x) (**H**) and neuropil-like islands (magnification 400x) (**I**). Diffuse expression of SOX10 by tumor cells (magnification 400x) (**J**), without immunopositivity for GFAP staining only residual astrocytes (magnification 400x) (**K**). Neurofilament staining confirming the diffuse growth pattern of the tumor (magnification 400x) (**L**). HPS: Hematoxylin phloxin saffron. Scale bars represent 50 μm

**Fig. 3:**
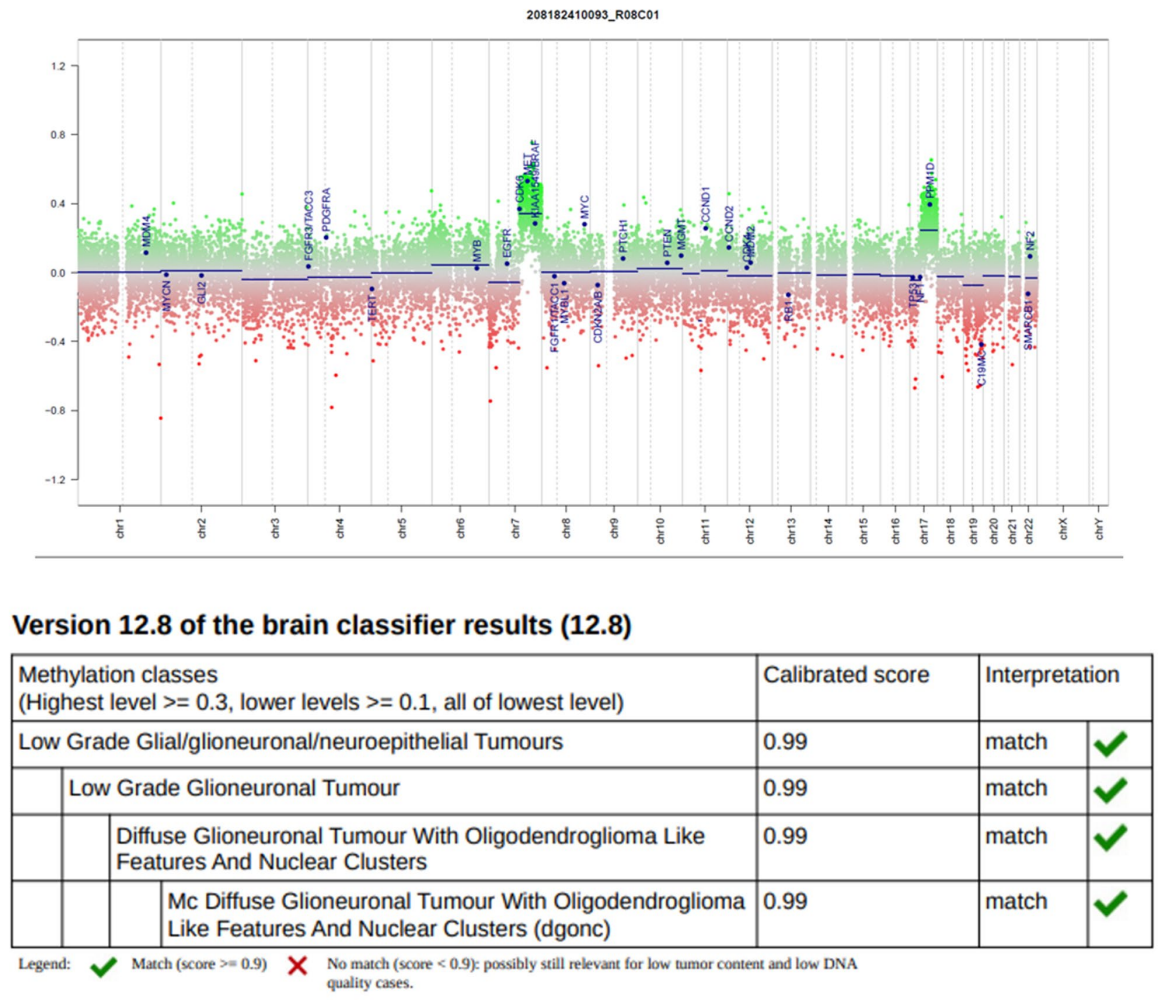
Results from DNA-methylation profiling analysis. Copy number variation analysis from DNA-methylation profiling analysis showed a partial gain of chromosome 7q and a gain of chromosome 17q and classified the tumor as a diffuse glioneuronal tumor with oligodendroglioma-like features and nuclear clusters

A previously healthy 11-year-old boy presented with an episode of generalized seizure. A computerized tomodensitometry scan of the head showed a slightly hyperdense, heterogeneous cortical mass with calcifications in the left central sulcus. Bone scalloping was observed next to the tumor (Fig. 1A). Cerebral magnetic resonance imaging showed the mass to have high signal intensity on T2-weighted images, heterogeneous contrast enhancement, high signal intensity on diffusion weighted images, with an intermediate apparent diffusion coefficient and low cerebral blood flow measured by arterial spin labeling perfusion (Fig. 1B-F). The mass was surgically excised. Microscopically, we observed a densely cellular proliferation, infiltrating the brain parenchyma (Fig. 2D). The tumor presented various histopathological patterns with pseudo-rosettes, spongioblastic, and oligodendroglioma-like features (Fig. 2A-C). Some tumor cells were multinucleated (Fig. 2B). Signs of malignancy were obvious (microvascular proliferation, a high mitotic index of 29 mitoses per 1.6 mm^2^, an elevated MIB-1 labeling index (30%) and necrosis (Fig. 2D-E). Microcalcifications were observed (Fig. 2F**)**. There were no perivascuclar lymphocytic infiltrations, xanthomatous cells, eosinophilic granular bodies or Rosenthal fibres. Immunohistochemistry revealed INI1, BRG1, H3K27me3 and ATRX to be maintained. The tumor cells diffusely expressed OLIG2, synaptophysin, MAP2, NeuN (not shown) and SOX10 but did not express GFAP (Fig. 2G-K). Synaptophysin revealed the presence of neuropil islands (Fig. 2I). Neurofilament staining confirmed the tumor’s diffuse growth pattern (Fig. 2L). No extravascular expression of CD34 was present. Lin28A, IDH1R132H and BRAFV600E were negative. DNA-methylation analysis classified the tumor as a DGONC (v12.8 with a calibrated score 0.99) (Fig. 3). The copy number variation analysis, generated from the DNA methylation profile, failed to reveal a chromosome 14 monosomy, but revealed a gain of chromosome 17q (Fig. 3). The DNA sequencing analysis found a *PIK3R1* mutation. The RNA-sequencing analysis failed to reveal any fusion.

## Discussion and conclusions

DGONC affect predominantly children (85% of reported cases) with a median age of 9 years-old (ranging from 2 to 75) without sex predominance (female to male ratio of 1.1) [1, 3–5]. They are found in supratentorial locations outside the ventricles [1, 3–5]. The tumors show variations in differentiation and cellularity, from moderately cellular oligodendroglioma-like features, microcalcifications and neuropil islands to highly cellular tumors, explaining why reported cases have previously been identified as primitive neuroectodermal tumors, anaplastic oligodendrogliomas, glioblastomas, extraventricular neurocytomas, or low-grade gliomas and dysembryoplastic neuroepithelial tumors [1, 3–5]. DGONC is a provisional tumor type and its classification among glioneuronal vs. embryonal tumors is debated [2]. Herein, we present for the first time a case harboring pseudo-rosettes. The variety of histopathology and grading (neurocytic from embryonal morphology with neuropil islands and spongioblastic features) in association with the co-expression of OLIG2 and neuronal markers may morphologically resemble CNS NB-FOXR2 [2, 6]. However, in contrast to the aforementioned tumors, DGONC expresses SOX10, which may potentially be important for a diagnosis [7]. A chromosome 1q gain, which is almost always observed in CNS NB-FOXR2, may also be encountered in DGONC (19/35 reported cases, 54%) [1, 5, 6]. The monosomy of chromosome 14, which is a desirable diagnostic criterion for the current WHO classification, may be exceptionally absent (1/44 reported cases, 2%) [1–5], as was the case in our observation. Because of its morphological heterogeneity and immunoprofile (co-expression of OLIG2 and synaptophysin), other differential diagnoses include glioneuronal tumors kinase-fused, glioneuronal tumors with an ATRX alteration, kinase fusion and anaplastic features (GTAKA), embryonal tumors with a *BRD4::LEUTX* fusion, CNS tumors with a *BCOR* alteration, pediatric diffuse high-grade gliomas, *IDH-* and H3-WT (with *MYCN* amplification), but also diffuse leptomeningeal glioneuronal tumors, polymorphous low-grade neuroepithelial tumors of the young, multinodular and vacuolating neuronal tumors, and extraventricular neurocytomas. All of these diagnoses can be ruled out by DNA-sequencing, RNA-sequencing, and DNA-methylation profiling analyses. Additional molecular features may be observed in DGONC, particularly *GNAS* mutations, a *PIK3CA* mutation [1], or *PIK3R1* mutations, as in our case.

In a case similar to ours, Benesch et al. [3] reported homogeneous imaging characteristics of nine DGONC, including sharply delineated cortical-subcortical supra-tentorial masses, with small mass effect, bone remodeling, intermediate diffusion restriction, and variable contrast uptake. These imaging features may resemble embryonal tumors, except for diffusion, which is less consistently restricted in DGONC, arguing radiologically for a less aggressive behavior. Very few clinical data are available concerning the outcome of DGONC. Data from the literature show that 4/22 (18%) cases presented a progression [1, 3–5] with a median progression-free survival of 18 months (ranging from 4 to 26) [1, 3] and only two patients were deceased from their disease at the end of follow-up (2/25 cases, 8%) [1, 3–5], 25 and 96 months after the initial diagnosis [1]. Interestingly, three cases were treated by total resection alone, and did not present any recurrence at the end of follow-up [3, 5], one of them 108 months after the initial diagnosis [3]. Further series are needed to determine if the different morphologies (well and poorly differentiated) are correlated to different outcomes.

In conclusion, we expanded the characterization of DGONC with a novel case, which does not harbor a chromosome 14 monosomy and presents histopathological features similar to an embryonal tumor. Further studies are needed to characterize in detail this rare type of tumor and to conclude its cellular origin and prognosis.
